# Alteration autonomic control of cardiac function during hemodialysis predict cardiovascular outcomes in end stage renal disease patients

**DOI:** 10.1038/s41598-019-55001-4

**Published:** 2019-12-11

**Authors:** Chih-Chin Kao, Chi-Ho Tseng, Men-Tzung Lo, Ying-Kuang Lin, Chien-Yi Hsu, Yueh-Lin Wu, Hsi-Hsien Chen, Feng-Yen Lin, Chen Lin, Chun-Yao Huang

**Affiliations:** 1 Division of Nephrology, Department of Internal Medicine, Taipei Medical University Hospital, Taipei, Taiwan; 2 Division of Nephrology, Department of Internal Medicine, School of Medicine, College of Medicine, Taipei Medical University, Taipei, Taiwan; 3 Graduate Institute of Clinical Medicine, College of Medicine, Taipei Medical University, Taipei, Taiwan; 4 Department of Biomedical Sciences and Engineering, National Central University, Taoyuan city, Taiwan; 5 Graduate Institute of Biomedical Electronics and Bioinformatics, National Taiwan University, Taipei, Taiwan; 6 Center for Biotechnology and Biomedical Engineering, National Central University, Taoyuan city, Taiwan; 7 Division of Nephrology, Department of Medicine, Landseed International Hospital, Taoyuan city, Taiwan; 8 Institute of Clinical Medicine, National Yang Ming University, Taipei, Taiwan; 9 Division of Cardiology, Department of Internal Medicine, School of Medicine, College of Medicine, Taipei Heart Institute, Taipei Medical University, Taipei, Taiwan; 10 Division of Cardiology and Cardiovascular Research Center, Department of Internal Medicine, Taipei Medical University Hospital, Taipei, Taiwan

**Keywords:** Cardiovascular diseases, Cardiovascular diseases, Haemodialysis, Haemodialysis

## Abstract

Dialysis-induced hemodynamic instability has been associated with increased risk of cardiovascular (CV) mortality. However, the control mechanisms beneath the dynamic BP changes and cardiac function during hemodialysis and subsequent CV events are not known. We hypothesize that the impaired hemodynamic control can be prognostic indicators for subsequent CV events in end stage renal diseaes (ESRD) patients. To explore the association of hemodynamic parameters and CV events in hemodialysis patients, we enrolled ESRD patients who received chronic hemodialysis without documented atherosclerotic cardiovascular disease and hemodynamic parameters were continuously obtained from the impedance cardiography during hemodialysis. A total of 35 patients were enrolled. 16 patients developed hospitalized CV events. The statistical properties [coefficient of variance (standard deviation / mean value; CoV)] of hourly beat-to-beat dynamics of hemodynamic parameters were calculated. The CoV of stroke volume (SV) and cardiac index (CI) between the 1^st^ and 2^nd^ hour of dialysis were significantly increased in patients without CV events compared to those with CV events. Higher CoV of SV_diff_ and CI_diff_ were significantly correlated with longer CV event-free survival, and the area under the receiver operating characteristic (ROC) curve showed fair overall discriminative power (0.783 and 0.796, respectively). The responses of hemodynamic control mechanisms can be independent predictive indexes for lower hospitalized CV events, which implies that these patients who have better autonomic control systems may have better CV outcomes.

## Introduction

The risk of cardiovascular (CV) mortality in dialysis patients is approximately 9 times higher than that of the general population^1^, and young dialysis patients were characterized by extraordinarily high risk^2^. More than half of the CV events are the result of fatal arrhythmia and congestive heart failure, and some are the result of myocardial infarction^3^. In addition to the already identified CV risks including hypertension, hyperlipidemia, diabetes^4^ and electrolytes imbalance^5^, the intradialytic hypertension/hypotension or autonomic instability were thought to worsen their CV outcome in dialysis patients. Dialysis-induced hemodynamic instability was one of the most common complications, and those patients with unstable hemodynamics during hemodialysis were associated with worse outcomes^6^. A large retrospective cohort showed that the modest decline of BP between initiation and the end of hemodialysis was accompanied by the most favorable outcomes^7^. The relationship between the pre- and post-hemodialysis BP changes and all-cause mortality in the end-stage renal disease (ESRD) patients was described as “U- or J-shaped associations with lowest risk around −20 mm-Hg between post- and pre-dialysis BP in two observational studies ”^8,9^. Furthermore, the greater fluctuation of systolic BP (SBP) measured at 30-min intervals during dialysis was shown to be associated with higher risk of all-cause mortality and CV mortality in these patients^10^.

The BP homeostasis is one of the most sophisticated control mechanisms that incorporates several systems interacting with each other continuously^6,11,12^. The relatively stable BP in a constantly changing environment is the physiologic response of continuously fine-tuning the hemodynamic variables including cardiac output [(stroke volume (SV) * heart rate (HR)] and systemic vascular resistance (SVR) by the underlying control mechanisms. Increased beat-to-beat BP variability is not only a sign of impaired control systems but also a risk factor for CV events in hypertensive patients^13,14^. In addition, evidence has shown that the dynamics of beat-to-beat SV or HR can serve as earlier precursors to fluid responsiveness for several critical conditions^15–17^ before the actual change of BP. The temporal changes of hemodynamic variables in patients undergoing dialysis can be regarded as how the control systems respond^18,19^ while being exposed to continuous fluid shifts and osmolarity changes. However, few studies focused on continuously monitoring the hemodynamic variables other than BP during hemodialysis, and the relationship between the alteration of cardiovascular systems during hemodialysis and CV events is yet to be reported. We hypothesize that the impaired hemodynamic control can be prognostic indicators for subsequent CV events in ESRD patients and the dynamics of the intradialytic hemodynamic parameters derived from impedance cardiography were quantified to explore the association of hemodynamic parameters and CV events in hemodialysis patients.

## Results

### Demographics of our patients

**Figure 1:**
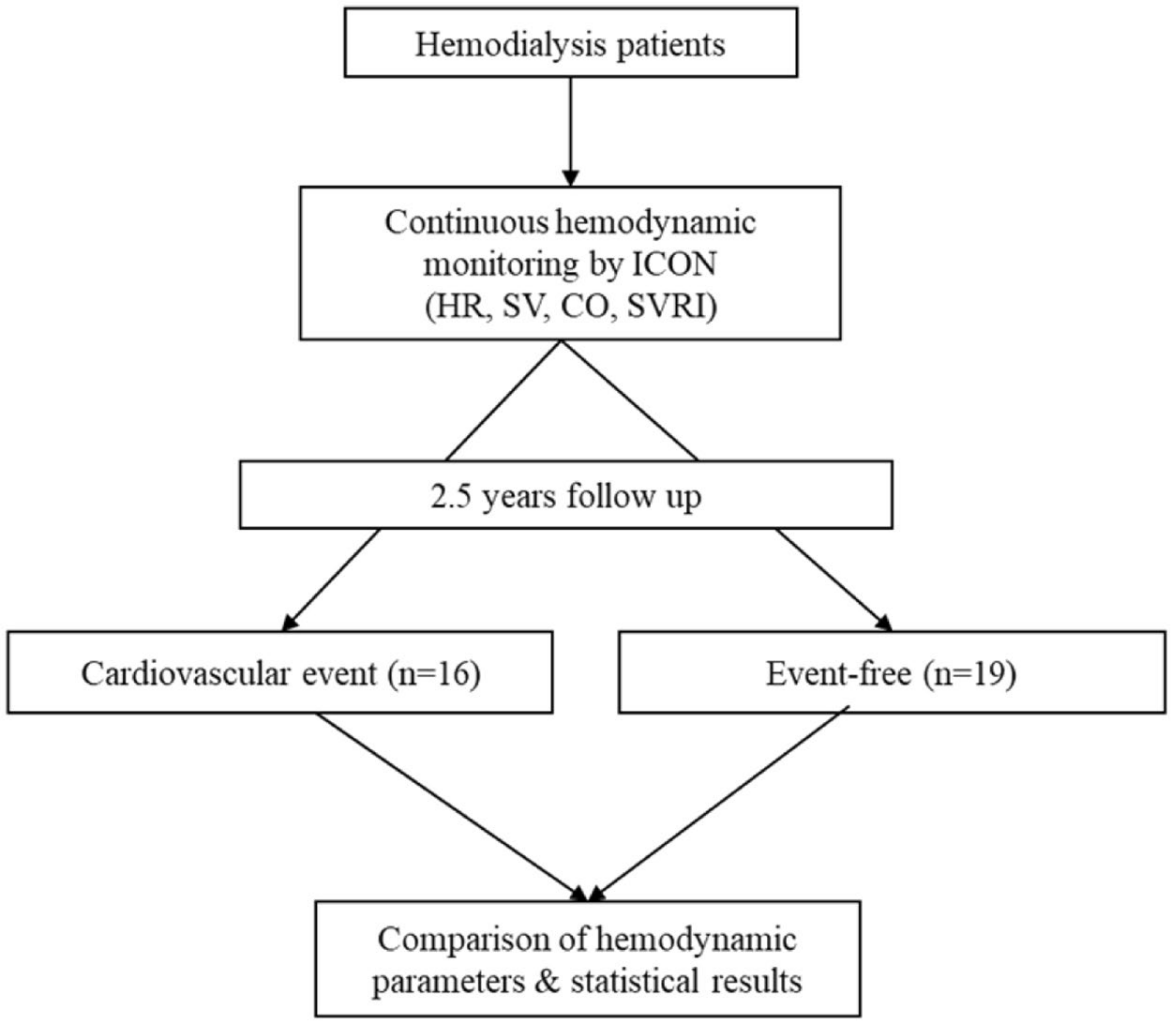
Flow chart of the study.

A total of 35 patients were enrolled, and the flow chart of this study is shown in Fig. 1. The mean age of our study objects was 57 ± 14 years and 24 (68.6%) were male. The mean follow-up duration was 531 ± 53 days for all patients, with a mean of 252 ± 56 days in the CV events group, and 765 ± 30 days in the non-CV events group. 16 (45.7%) of them developed CV events, and the remaining patients were event-free until the study end. The demographics are shown in Table 1. The prevalence of comorbidities were not different between groups, except insulin-dependent diabetes mellitus. Biochemistry results were similar except for the higher potassium level in the non-CV events group. The hemodialysis parameters and BP at the start and end of dialysis were not significantly different. The 16 CV events were 8 for MACE (cardiac death n = 5, myocardial infarction n = 1, ischemic stroke n = 2) and 8 for hospitalization for a cardiovascular-related illness (heart failure n = 3, symptom-driven revascularizations n = 4, acute limb ischemia n = 1) (Table 2).

**Table 1:** Demographics of patients according to CV events or not.

Parameter	All	No CV events (n = 19)	CV events (n = 16)	P
Age	57 ± 14	52 ± 12	63 ± 14	0.017
Sex (male, %)	24 (68.6%)	12 (63.2%)	12 (75.0%)	0.452
HD vintage (years)	6.3 ± 6.8	4.6 ± 4.2	8.2 ± 8.8	0.128
Smoking	8 (22.9%)	3 (15.8%)	5 (31.3%)	0.278
BMI	24.1 ± 4.0	23.5 ± 4.2	24.8 ± 3.7	0.348
DM	14 (40%)	5 (26.3%)	9 (56.3%)	0.072
HTN	25 (71.4%)	16 (84.2%)	9 (56.3%)	0.068
Hyperlipidemia	20 (57.1%)	8 (42.1%)	12 (75.0%)	0.050
**Medications**				
Aspirin	7 (20%)	3 (15.8%)	4 (25.0%)	0.497
Clopidogrel	7 (20%)	1 (5.3%)	6 (37.5%)	0.018
ACEI/ARB	15 (42.9%)	11 (57.9%)	4 (25.0%)	0.050
Beta blockers	12 (34.3%)	6 (31.6%)	6 (37.5%)	0.713
CCB	13 (37.1%)	8 (42.1%)	5 (31.3%)	0.508
Midodrine	6 (17.1%)	2 (10.5%)	4 (25.0%)	0.258
Statins	7 (20%)	3 (15.8%)	4 (25.0%)	0.497
OHA	5 (14.3%)	1 (5.3%)	4 (25.0%)	0.096
Insulin	6 (17.1%)	1 (5.3%)	5 (31.3%)	0.042
**Laboratory**				
Albumin	4.0 ± 0.3	4.1 ± 0.3	3.9 ± 0.3	0.118
BUN	71 ± 15	71 ± 13	71 ± 17	0.963
Cr	11.9 ± 2.2	12.1 ± 2.0	11.7 ± 2.4	0.573
K	4.8 ± 0.7	5.1 ± 0.7	4.6 ± 0.7	0.042
Ca	9.2 ± 0.9	9.0 ± 0.9	9.4 ± 0.9	0.217
P	5.4 ± 1.7	5.5 ± 1.6	5.3 ± 1.9	0.796
Hb	11.2 ± 1.2	11.4 ± 1.0	11.0 ± 1.5	0.311
HbA1c	7.6 ± 2.0	6.6 ± 2.1	8.2 ± 1.9	0.189
PTH	348 ± 299	414 ± 317	270 ± 264	0.158
**Hemodialysis parameters**				
UFR	0.7 ± 0.3	0.8 ± 0.4	0.7 ± 0.3	0.580
Duration	3.9 ± 0.3	3.9 ± 0.2	3.9 ± 0.3	0.549
Mean Qb	259 ± 28	258 ± 21	261 ± 35	0.747
SBP_start	146 ± 34	154 ± 32	138 ± 34	0.164
DBP_start	80 ± 18	82 ± 18	78 ± 20	0.495
HR_start	76 ± 10	75 ± 9	78 ± 11	0.505
SBP_end	143 ± 29	146 ± 25	140 ± 35	0.572
DBP_end	86 ± 19	89 ± 17	83 ± 20	0.408
HR_end	78 ± 15	78 ± 18	78 ± 12	0.926

ACEI/ARB: angiotensin-converting enzyme inhibitor/angiotensin II receptor blocker, BMI: body mass index, CCB: calcium channel blocker, Cr: creatinine, CV: cardiovascular, DBP: diastolic blood pressure, HDM: diabetes mellitus, b: hemoglobin, HD: hemodialysis, HR: heart rate, HTN: hypertension, OHA: oral hypoglycemic agents, PTH: parathyroid hormone, SBP: systolic blood pressure, Qb: blood flow per minute, UFR: ultrafiltration rate.

**Table 2:** The subtypes of cardiovascular (CV) events.

CV events type	N (%)
MACE	**8 (50.0%)**
Cardiac death	5 (31.3%)
Myocardial infarction	1 (6.3%)
Ischemic Stroke	2 (12.5%)
Hospitalization for a cardiovascular-related illness	**8 (50.0%)**
Heart failure	3 (18.8%)
Symptom-driven revascularizations	4 (25.0%)
Acute limb ischemia	1 (6.3%)

CV: cardiovascular; MACE: major adverse cardiovascular events.

### Continuous hemodynamics monitoring and their effects on CV outcomes

**Figure 2:**
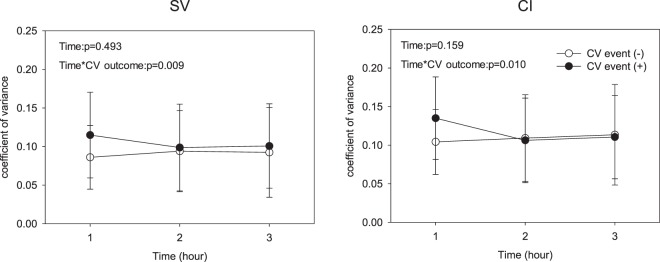
Temporal changes of coefficient variance of (**a**) stroke volume (SV) and (**b**) cardiac index (CI) during dialysis between groups.

For patients with or without CV outcome, the hourly mean SV gradually dropped over the course of dialysis [F(2, 66) = 4.397, p = 0.032], while the hourly mean HR[F(2, 66) = 0.708, p = 0.496], CI [F(2, 66) = 0.285, p = 0.697], and SVRI [F(2, 66) = 0.744, p = 0.431] were not significantly changed. In addition, the direction changes of hourly CoV of SV [F(2, 66) = 5.42, p = 0.009] and CI [F(2, 66) = 4.891, p = 0.010] in the early course of hemodialysis were significantly different between the two groups **(**Fig. 2**)**.

### Reduced CoV of SV and CI in early course of dialysis associated with poor CV outcome

**Figure 3:**
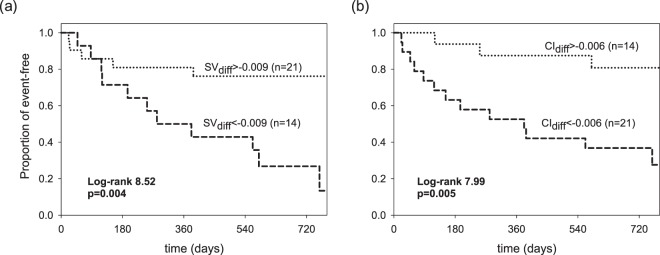
Survival analysis of CV events according to the optimal dichotomized threshold of differences of coefficient variance of (**a**) SV between 2^nd^ and 1^st^ hr of dialysis (SV_diff_) and (**b**) CI between 2^nd^ and 1^st^ hr of dialysis (CI_diff_).

**Figure 4:**
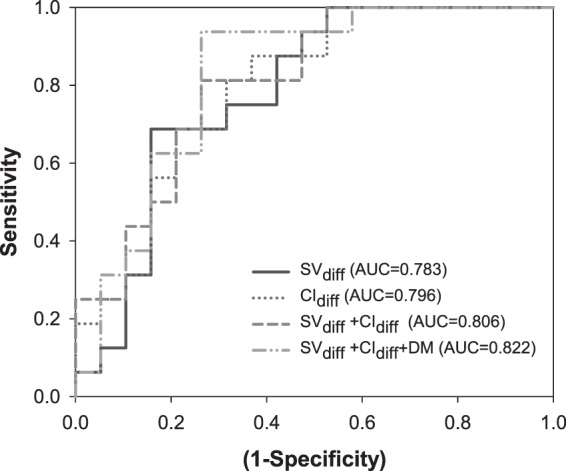
Receiver operating characteristic curves for the differences of coefficient variance of SV between 2^nd^ and 1^st^ hr of dialysis (SV_diff_; solid line), CI between 2^nd^ and 1^st^ hr of dialysis (CI_diff_; dotted line) and the multivariable generalized linear model with binomial distribution on the combination of SV_diff_ and CI_diff_ (gray dashed line) and combination of SV_diff_, CI_diff_, and DM (gray dash-dotted line). Abbreviations: CI: cardiac index, DM: diabetes mellitus, SV: stroke volume.

In multivariate analysis, higher CoV of SV_diff_ (HR = 0.954, 95% CI: 0.924–0.984, p = 0.003) and CI_diff_ (HR = 0.985, 95% CI: 0.972–0.998, p = 0.026) were independent predictors of better prognosis in these patients **(**Table 3**)**. In addition, history of diabetes mellitus was a prognostic indicator. The optimal cut point of CoV of SV_diff_ and CI_diff_ were −0.009 and −0.006 (40^th^ and 60^th^ percentile of the corresponding variables), which for the most part predicted the differences in survival curves. Figure 3 depicted the Kaplan-Meier survival curves of the CoV of SV_diff_ and CI_diff_ with the dichotomized threshold and higher CoV of SV_diff_ and CI_diff_ correlated with longer event-free survival (p = 0.004 and p = 0.005, respectively). In ROC analysis, the AUCs of the CoV of SV_diff_ (0.783) and CI_diff_ (0.796) showed fair overall discriminative power in predicting patents with poor CV outcome. Added-on diabetes with CoV of SV_diff_ and CI_diff_ increased little discriminative power with an AUC of 0.822 **(**Fig. 4**)**.

**Table 3:** Risk of CV events according to the clinical variables and the differences of coefficient variance of SV and CI between 2^nd^ and 1^st^ hour of dialysis (SV_diff_ and CI_diff_).

Variable	Univariate	P	Multivariate	P
SV_diff_	0.958 (0.933–0.984)	0.002	0.954 (0.924–0.984)	0.003
CI_diff_	0.984 (0.968–0.996)	0.011	0.985 (0.972–0.998)	0.026
Age	1.048 (0.993–1.107)	0.086	1.055 (0.853–1.305)	0.623
Sex	0.512 (0.131–1.996)	0.335	1.580 (0.71–35.070)-	0.772
BMI	1.016 (0.881–1.172)	0.828	0.902 (0.742–1.097)	0.302
Smoking	2.123 (0.529–8.517)	0.288	0.094 (0.001–232.28)	0.553
DM	6.433 (1.666–24.834)	0.007	5.611 (1.277–24.658)	0.022
HTN	0.550 (0.157–1.926)	0.350	0.147 (0.008–2.881)	0.207
Hyperlipidemia	2.298 (0.641–8.231)	0.201	7.019 (0.039–1247.4)	0.461
K	0.366 (0.144–0.930)	0.035	0.106 (0.001–10.361)	0.337
Ca	0.933 (0.331–2.629)	0.896	0.984 (0.042–23.122)-	0.992
P	0.668 (0.401–1.114)	0.668	1.649 (0.328–8.282)-	0.544

Multivariate forward-step model: adjusted for age, sex, BMI, potassium, Calcium, and phosphate levels, diabetes, hypertension, hyperlipidemia, smoking. BMI: body mass index, CI_diff_: coefficient variance of cardiac index (between 2^nd^ and 1^st^ hr of dialysis), CV: cardiovascular, DM: diabetes mellitus, HTN: hypertension, SV_diff_: coefficient variance of stroke volume (between 2^nd^ and 1^st^ hr of dialysis).

## Discussion

Decrease in blood volume during the course of hemodialysis inevitably elicited compensatory mechanisms responding to a hemodynamic challenge. This hemodynamic instability was often a manifestation of the deteriorated control system particularly in the elderly^14^, and patients with poor cardiac function^20^. However, the clinical meaningful events of hemodynamic instability were presented by intradialytic or pre/post-dialytic BP changes, and we often ignored the underlying dynamics of the control mechanisms. In this study, we applied the dynamics of several hemodynamic parameters on a beat-by-beat basis, including HR, SV, CI and SVRI recorded from an impedance cardiograph, to explore the underlying mechanisms. The main findings are that the enhanced regulation of SV and CI in the early course of hemodialysis is associated with better CV outcomes in ESRD patients.

Longitudinal studies found that greater BP variability at pre-dialysis was not only associated with increased risk of all-cause mortality^21^ but also with cardiac structural changes such as left ventricular hypertrophy in chronic hemodialysis patients^22^. This implies that the declining homeostatic control mechanisms may be associated with poor prognosis. The impairment of homeostatic control mechanisms can contribute to larger differences in pre-to-post dialytic BP^8,9^ and the increased intra-dialytic BP measured during hemodialysis^10^. However, the mechanisms of increased BP variability are not well known. The compensated mechanisms in response to rapid fluid shifting during hemodialysis included increased CI or increased SVRI^18,19^. In this study, the patients presented gradual reduction of hourly SV during dialysis while maintaining relatively stable HR, CI, and SVRI. This may indicate that the average values of the hemodynamic parameters can be insensitive to the variability or dynamics generated from the physiological systems as suggested by several studies on HR and BP variability^13,23,24^.

In hypertensive patients, the variability of diastolic BP continuously recorded by arterial line quantified at 30-minute intervals was increased significantly in hypertensive patients compared to normotensive subjects, but the CoV of continuous systolic and diastolic BP over the long term (24 hours) or short term (30 minutes) significantly decreased in hypertensive patients^25^. CoV is a normalization function that estimates the ratio of SD to its mean. Since a higher CoV represents a greater percentage of variability irrespective of the different mean values, it can avoid the potential issue related to the larger mean values with greater SD in physiological signals. That is, increased BP variability can result from elevated BP, but lower CoV reflects declining control mechanisms such as decreased baroreflex sensitivity^26^ and autonomic system dysfunction^24,27^. In the early course of hemodialysis, the patients with increased hourly CoV of SV and CI between the 1^st^ and 2^nd^ hour were associated with better CV outcomes, while hourly SD of the hemodynamic parameters showed no associations. In addition, the patients with unstable BP had lower CoV of SVRI and CI during hemodialysis compared to those with stable BP^28^. Properly enhancing the regulatory responses during hemodialysis can be a sign of better underlying control systems. A study that enrolled 109 patients showed worsening of left ventricular diastolic function early during hemodialysis session^29^. The mean early diastolic velocity (e’) change at 60 minutes of dialysis was not correlated with volume indexes. It may underlie the non-volume related mechanisms involved during early hours of hemodialysis. This may explain why the CoV change in SV and CI were only found in the early course of hemodialysis.

More recently, Yoshihara F *et al*.^30^ defined the stroke volume variation (SVV) recorded by impedance cardiography between the maximal and minimal SV over 10 consecutive beats to assess the SV dynamics during dialysis. The study showed that higher SVV is an independent predictor of unstable BP (decreased mean arterial pressure over 10 mmHg). Numerous studies in the non-dialysis population have shown that higher respiratory-related SVV during surgery^16^ or hypovolemia^31^ can be an indicator of inadequate blood volume. The discrepancies between hourly CoV of SV and SVV may arise from the SVV quantifying the mechanisms related to certain time scales such as respiration-driven SV fluctuations and averaging them over the course of dialysis. The hourly CoV of SV could contain additional mechanisms and their hourly changes together. Most patients developed intra-dialytic hypotension due to blood volume decrease without timely refills. A study showed that patients with post-dialysis hypotension were related to an altered response in peripheral resistance but not a change in cardiac performance^32^. This may explain why higher SVV can predict unstable BP. However, Titapiccolo *et al*.^24^ reported that renal failure patients with peripheral vascular disease had reduced cardiac baroreflex and reduced sympathetic activity. Several studies also proposed BP variability might be due to less control of peripheral vascular resistance by reduced cardiac baroreflex during volume depletion^24,26^. Moreover, insulin-dependent diabetes itself is an independent risk factor for CV events, which could be partly attributed to the autonomic neuropathy that consequently cause higher risk of arrhythmia, QT prolongation^33^, and impaired BP control mechanisms^34^. This warrants further studies on exploring the temporal changes of the hemodynamic parameters during dialysis to further clarify the linkage of specific underlying mechanisms to the dynamical patterns. Nevertheless, we demonstrate that the dynamics of the control mechanisms during the early course of dialysis is crucial. Poor control of the hemodynamic variables is a risk factor for CV events.

Several limitations are present in this study. First, our results only demonstrated the association. The causal effect of BP, hemodynamic parameters on the CV outcomes may only be established by randomized controlled trials. Second, this non-invasive device was not correlated with other fluid measurement devices, such as a bioimpedance spectroscopy device, or cardiac sonography as a validation. The volume status is pre-load, which is also an important parameter influencing the SV. Fluid management depends on the physical examination of edema status and absence of intra-dialytic hypotension. This volume status and ultrafiltration rate may affect the heart function as well. Third, our study was a single center design, and the sample size is small. Monitoring only one hemodialysis session may be confounded by the variability in volume and condition, although we chose the mid-week session to avoid this. In addition, a vast number of covariates were corrected in the model, though it may induce a type I error. We also did not record the changes of dialysate temperature, though whether it affect the MACE or all-cause mortality may need more evidences^35^. Further validation cohorts with a larger sample size or increased hemodialysis session observations are needed to overcome this and ensure the findings.

In conclusion, change in SV and CI in the early period of dialysis may have predictive values for CV outcomes in hemodialysis patients, which implies that patients with better autonomic control systems may have better CV outcomes.

## Materials and Methods

### Study population

The observation, prospective cohort was performed from May 2015 to May 2018. Patients were recruited from the dialysis center of Taipei Medical University Hospital. The inclusion criteria were patients with ESRD receiving regular hemodialysis three times weekly for more than 3 months. The exclusion criteria were patients who were suffering acute illness or in hospital with documentation of atherosclerosis cardiovascular disease, aged older than 80 or younger than 20. This study was approved by the Institutional Review Board of Taipei Medical University (N201404050) and written informed consent was obtained from each participant before enrollment in this study. Furthermore, all methods were performed in accordance with the approved guidelines.

### Study design and procedures

Patients’ clinical parameters, co-medications, and biochemistry results were collected. Risks for cardiovascular disease were recorded, including smoking, body mass index, hypertension, diabetes mellitus and hyperlipidemia. In addition, the hemodialysis parameters including blood flow, dialysate calcium concentration, dialysis duration and ultrafiltration rate were recorded. After enrollment, we chose the mid-week dialysis session as the observation period. During hemodialysis, BP was measured with an electric sphygmomanometer in the supine position at 10-min intervals starting from 10 minutes before initiation of hemodialysis, and it was ended 10 minutes after the end of hemodialysis. Systemic vascular resistance indexes (SVRI) were calculated as the ratio of mean arterial pressure to cardiac index (CI, cardiac output divided by body surface area) to normalize the differences in patients’ size. Continuous impedance cardiography was recorded and the beat-to-beat hemodynamic parameters including HR, SV, CI were derived from the ICON^®^ machine (Electrical Cardiometry (EC™), Osypka Medical, Inc., CA, USA). The definition of HR is the rate at which the heart beats; SV is the volume of blood pumped from the ventricle of the heart in one beat; CI is the measure of cardiac output per square meter of body surface area^36^. This non-invasive method was described for hemodynamic monitoring^37^. Analysis was performed on an hourly basis, and several statistical properties of those hemodynamic parameters such as the mean value, standard deviation (SD), and coefficient of variance (CoV) were carried out to explore the dynamical changes of these parameters during dialysis. The measurement of “CoV” was calculated as the ratio of SD to the mean, which represents the degree of variability in proportion to its mean, and a higher CoV indicates better control of the homeostatic system^25^. The trend of these hemodynamic parameters were described and the difference of coefficient variance between hourly-based changes of SV and CI were studied (SV_diff_ and CI_diff_).

### CV events

We prospectively followed these patients until the occurrence of CV events or the end of study in May, 2017. The prespecified CV events were defined according to the primary diagnosis of the discharge note. The CV events included the composition of major adverse cardiovascular events (MACE) (cardiac death, myocardial infarction and ischemic stroke) or hospitalization for a cardiovascular-related illness, including heart failure, symptom-driven revascularizations, and acute limb ischemia.

### Statistical analysis

Baseline demographic characteristics are represented as the mean ± SD for continuous variables, and as proportions for categorical variables. The between-group comparisons of categorical variables were calculated by χ^2^ or Fisher’s exact test. Hourly based parameters were compared by a 2 × 3 two-way ANOVA with repeated measures to examine the time course of hemodialysis and the outcome on the changes in hemodynamic parameters. The multivariate Cox model was performed, adjusted for age, gender, BMI, underlying diseases (e.g., diabetes mellitus, hypertension, and hyperlipidemia), smoking, and electrolyte (potassium, calcium, and phosphate) to evaluate the effects of the hemodynamic parameters on outcome. Furthermore, the optimal cut point of each selected variable was determined by the maximal hazards ratio of the dichotomized threshold calculated from the values between the 25^th^ to 75^th^ percentile with a 5 percentile step. The Kaplan-Meier survival analysis and log-rank analysis were performed to test whether the event-free probabilities over time of the stratified groups were significantly different. The receiver operating characteristic (ROC) curve of the selected variables were constructed, and generalized linear model with binary regression were applied to combine multiple variables. The areas under the ROC curve (AUC) were calculated to evaluate the overall predictive power. All statistical analyses were performed by using R software, version 3.5.0. and IBM SPSS Statistics for Windows Version 22 (IBM Corp. Released 2013. Armonk, NY:IBM Corp). A p value of <0.05 was considered statistically significant.

### Ethics approval and consent to participate

This study was approved by the Institutional Review Board of Taipei Medical University (Approval no. 201404050).

### Consent for publication

Written informed consent was obtained from all patients.

## Data Availability

All data related to this article are shown in the manuscript or are available upon request from the corresponding authors.
